# Exploring a role for community pharmacists in the identification of alcohol-related liver disease: a qualitative interview study with professionals, patients, and the public

**DOI:** 10.1093/alcalc/agae069

**Published:** 2024-10-05

**Authors:** Alexander Smith, Ryan M Buchanan, Julie Parkes, Kinda Ibrahim

**Affiliations:** School of Primary Care, Population Sciences and Medical Education, Faculty of Medicine, University of Southampton, Tremona Road, Southampton SO16 6YD, UK; National Institute for Health and Care Research Applied Research Collaboration Wessex, Southampton Science Park, Innovation Centre, 2 Venture Road, Chilworth, Southampton SO16 7NP, UK; University Hospital Southampton NHS Trust Department of Hepatology, Tremona Road, Southampton SO16 6YD, UK; School of Primary Care, Population Sciences and Medical Education, Faculty of Medicine, University of Southampton, Tremona Road, Southampton SO16 6YD, UK; University Hospital Southampton NHS Trust Department of Hepatology, Tremona Road, Southampton SO16 6YD, UK; National Institute for Health and Care Research Southampton Biomedical Research Centre, University of Southampton and University Hospital Southampton NHS Trust, Tremona Road, Southampton SO16 6YD, UK; School of Primary Care, Population Sciences and Medical Education, Faculty of Medicine, University of Southampton, Tremona Road, Southampton SO16 6YD, UK; National Institute for Health and Care Research Applied Research Collaboration Wessex, Southampton Science Park, Innovation Centre, 2 Venture Road, Chilworth, Southampton SO16 7NP, UK; School of Primary Care, Population Sciences and Medical Education, Faculty of Medicine, University of Southampton, Tremona Road, Southampton SO16 6YD, UK; National Institute for Health and Care Research Applied Research Collaboration Wessex, Southampton Science Park, Innovation Centre, 2 Venture Road, Chilworth, Southampton SO16 7NP, UK

**Keywords:** alcohol-related liver disease, identification, case-finding, community pharmacy, pharmacists, qualitative

## Abstract

**Aims:**

To explore the views and attitudes of professionals, patients and the public to a role for community pharmacists in the identification of alcohol-related liver disease (ArLD).

**Methods:**

Semi-structured interviews were conducted with a purposive sample of patients with ArLD, members of the public, pharmacy staff, and clinicians managing patients with ArLD across the Wessex region of south England. The interviews explored experiences of alcohol, ArLD and health advice in pharmacies and elicited views of what a pharmacist role in identifying ArLD could entail and factors influencing this. Transcripts were analysed using reflexive thematic analysis.

**Results:**

Twenty-six participants were interviewed and three themes were generated: (i) acknowledging, seeking help and engaging with a hidden problem; (ii) professional roles, boundaries and attributes; (iii) communication, relationships, collaboration and support. Participants reported key challenges to identifying people at-risk of ArLD. Offering testing for ArLD was perceived to motivate engagement but there were concerns about pharmacists performing this. A role was mostly seen to be finding people at-risk and engaging them with further care such as referral to liver services. This was perceived to require developing interprofessional collaborations, remuneration and training for pharmacy staff, and community-based liver testing.

**Conclusions:**

Professionals, patient and public participants recognized a role for pharmacists in the identification of ArLD. This was envisaged to incorporate educating pharmacy users about ArLD risk, and identifying and directly engaging those at-risk with liver and support services through development of interprofessional collaborations. The findings of this study support and can inform future work to develop this role.

## Teaser

**Short Summary:** A role for community pharmacists in ArLD identification was mostly viewed to be one of finding people at risk of ArLD, educating about ArLD and engaging people at-risk with further care. Interprofessional collaborations, remuneration and training for pharmacy staff, and community-based liver testing were viewed essential for such a role.

## Introduction

Alcohol is one of the leading risk factors for population health across the world (World Health Organization 2018). An estimated 3 million deaths per year worldwide are caused by harmful alcohol use, corresponding to 5.3% of all global deaths (World Health Organization 2018). In England, over 80% of alcohol-related deaths are due to liver disease (Office for National Statistics 2022). Alcohol-related liver disease (ArLD) affects those of middle age, with over 90% of deaths occurring in those under 75 years of age (Office for National Statistics 2022). In this group the number of deaths from ArLD has risen by 61.3% since 2003 (Office for Health Improvement and Disparities 2024).

To help address this problem there have been international calls for the earlier identification of ArLD using case-finding approaches (Karlsen et al. 2022; Allison et al. 2023). A case-finding approach for ArLD relies on identifying people who are at risk of ArLD from how much they drink and engaging them with testing to assess their liver for scarring (known as liver cirrhosis) (Karlsen et al. 2022). Risk of ArLD can be assessed through establishing number of standard drinks of alcohol consumed per week and/or through use of alcohol screening tools such as the alcohol use disorder identification tool for which thresholds for testing for ArLD have been described in international guidance (Newsome et al. 2017; Thursz et al. 2018).

There is a body of research indicating community pharmacists can undertake alcohol screening and provide brief interventions (Hattingh and Tait 2018) with pharmacists’ accessibility, particularly in areas of higher deprivation where alcohol-related harm is greatest, a well-recognized advantage (Todd et al. 2015). There may also be a higher prevalence of ArLD risk in people attending community pharmacy compared to the general population (Smith et al. 2021). Furthermore, pharmacists are able to take an active role in the identification of common chronic diseases by assessing risk and linking patients to appropriate healthcare providers (Ayorinde et al. 2013). However, there is a lack of research exploring the potential for community pharmacists to have a role in identifying ArLD in people with alcohol misuse (Smith et al. 2021).

To explore a potential role for community pharmacists in the identification of ArLD, we present a qualitative study that examines the views and attitudes of professionals, patients, and the public. Using an ethnographic approach, we aimed to gain an understanding of perceptions of such a role, what it could look like and potential barriers and facilitators to it by drawing on existing, contextualized experiences of participants.

## Method

Our study used semi-structured interviews given the potentially sensitive subject area and to provide flexibility around participants’ availability and avoid hierarchical influences that may be present if using focus groups (Ritchie and Lewis 2003). Topic guides were developed by A.S., refined with R.B. and K.I. and piloted with two patient and public involvement contributors (neither were participants in the study). The guides were iteratively revised as new concepts emerged during data collection and covered a number of areas including: experiences of community pharmacies providing health services and advice; experiences of existing alcohol and liver disease care in community pharmacy and healthcare in general; views on a role for pharmacy staff identifying ArLD including what this could entail and how this could link with existing care.

Purposive sampling was used to get a range of participants anticipated to provide the most useful and relevant data to achieve the study aims (Campbell et al. 2020). Heterogeneity of participants was desired to increase transferability of findings (Robinson 2014). Participants were recruited from the Wessex region of south England.

Professional participants incorporated community pharmacy staff (pharmacists and pharmacy assistants) and clinicians involved in the identification and care of patients with ArLD. A range of years of experience was sampled given the changing landscape of both community pharmacy and liver disease management in the last 20 years. Recruitment of pharmacy staff was through a Local Pharmaceutical Committee (LPC)—the local organizations for community pharmacies in England, each representing pharmacy owners in a defined locality. A study advert was placed on the LPC website and the LPC sent participant information sheets and an invitation for participation to pharmacies in their locality. For clinician participants we recruited key informants, using gatekeepers (hepatology consultants known to the research team) to identify and offer participation to other clinicians perceived to be information rich (Palinkas et al. 2015).

Patient and public (PP) participants included patients with lived experience of ArLD as well as pharmacy-using members of the public. For PP participants a range of age, sex and level of socioeconomic deprivation were desired given these are factors influencing outcomes of ArLD (Roerecke et al. 2019; Probst et al. 2020). Patients were recruited from liver outpatient clinics of a tertiary referral hospital through invitations from their clinicians. Members of the public were recruited through posters advertising study participation displayed in five pharmacies in the LPC locality, shared on social media (Twitter), and shared with an existing liver research-interested public group. Snowball sampling was also used whereby recruited participants were provided participant information sheets to pass onto any eligible contacts they may have (Palinkas et al. 2015).

Written consent was given by participants prior to being interviewed. Ethical approval was granted by University of Southampton Faculty of Medicine Ethics Committee (reference 64,726) and South Central—Oxford B Research Ethics Committee (reference 22/SC/0222). Interviews were conducted between September 2022 and August 2023 by A.S., a male doctoral researcher and hepatology specialty registrar with training in qualitative methods. A.S. was described as a liver research fellow to participants. Interviews were conducted either by telephone, video call on Microsoft Teams (version 1.5.00), or face-to-face in a private room of a community pharmacy or clinical research facility. Field notes were written following each interview. All interviews were audio recorded and transcribed before being checked for accuracy and anonymized by A.S.

Analysis was performed using thematic analysis based on the reflexive approach described by Braun and Clarke (Braun and Clarke 2022). We aimed to achieve data saturation, defined here as the point where no new themes are developed in the analysis, recognizing that the precise number of participants required to achieve saturation prior to analysis cannot be known (Braun and Clarke 2019). Thematic analysis can produce unanticipated insights, matching the exploratory aims of this work. Additionally the results are accessible to both the general public and healthcare professionals, important as our study will inform ongoing complex intervention development work involving these groups (Braun and Clarke 2006).

A.S. performed the analysis supported by K.I., an experienced qualitative researcher. The analysis was undertaken iteratively, moving back and forth between phases. Transcripts were imported into NVivo (release 1.6.1) and coded inductively. After coding four transcripts, a list of all codes generated was reviewed and refined to create a codebook. Two of these transcripts were separately coded by K.I. with coding subsequently discussed to share perspectives and interpretations of the data. The codebook was used to code further transcripts whilst allowing for new codes to be generated and existing codes to be refined. If new codes were generated, previously coded transcripts were re-examined for these codes. Notes were made of potential clusters of codes and themes during this process and regular meetings were held between A.S. and K.I. throughout to discuss coding and development of themes.

Following theme development, coded data extracts within themes and sub-themes were examined and revised until each theme was coherent and did not appear to overlap with another. The themes presented common patterns and important information reported by participants.

## Results

A total of 26 participants were recruited and interviewed; 15 professional participants and 11 PP participants. Table 1 shows a summary of participant characteristics. Most interviews were done remotely using Microsoft Teams (n = 12) or telephone (n = 8). Interviews lasted between 18 and 72 min with a median length of 39 min.

**Table 1:** Characteristics of interviewed participants

Characteristic	Group	
	Professionals (n = 15)	Patients and public (n = 11)
Age years; median (range)	48 (24–61)	56 (43–80)
Sex		
Female	11 (73)	4 (36)
Male	4 (27)	7 (64)
IMD Quintile		
1	-	2 (18)
2	-	4 (36)
3	-	0 (0)
4	-	1 (9)
5	-	4 (36)
Profession		-
Community pharmacy staff	8 (53)	
Pharmacist	4 (27)	
Pharmacy assistant	4 (27)	
Clinician managing ArLD	7 (46)	
Consultant in	2 (13)	
gastroenterology and hepatology		
Hepatology nurse specialist	2 (13)	
Fibroscan technician	1 (7)	
GP	2 (13)	
Years of experience in current role; median (range)	12 (.5–28)	-
Lived experience of ArLD	-	6 (54)
Ethnicity		
White British	-	10 (91)
White Irish	-	1 (9)

Numbers are counts (percentage) or where stated median with range

IMD index of multiple deprivation, GP general practitioner, ArLD alcohol-related liver disease

Three overarching themes emerged from the analysis with each theme containing a number of sub-themes as summarized in Table 2. The analysis is described according to these themes and sub-themes with illustrative quotes from participants (labelled as participant number/participant type/age and sex) to enhance this description.

**Table 2:** Summary table of themes and subthemes

**Theme and theme description**	**Sub-themes**
*Acknowledging, seeking help and engaging with a hidden problem* This theme incorporates participants views and experiences around how alcohol-related health problems are realized and the challenges relating to this. Perceptions around engaging patients with possible alcohol-related health problems with a process of assessment, identification and ongoing care area are also examined.	*Stereotyping and self-awareness of drinking* *Seeking advice and revealing hidden conditions* *Stigma, honesty and routinely contextualizing* *Enabling and facilitating motivated engagement*
*Professional roles, boundaries and attributes* This theme examines the experiences of health advice in community pharmacy in relation to alcohol-related health problems. It further explores views and perceptions of what role community pharmacy staff could play in identification of ArLD alongside other healthcare professionals and perspectives of the attributes of both pharmacy staff and the community pharmacy environment that may impact such a role.	*General health, alcohol and liver disease advice in community pharmacy* *Perceived abilities of community pharmacists for a role in ArLD identification* *Bypassing GPs* *Benefits and challenges of the community pharmacy setting* *Optimizing a service model of delivery in pharmacy*
*Communication, relationships, collaboration and support* This theme explores views regarding the links and communication between community pharmacy and other healthcare professionals. Further perceptions of needs in relation to this and also in relation to the wider interdisciplinary, collaborative care of patients with possible ArLD are also considered.	*Making referrals and pathways simple, clear and efficient* *Two-way interprofessional communication* *Establishing relationships and collaborating* *Unmet support needs*

*ArLD; alcohol-related liver disease; GP; general practitioner*

### Acknowledging, seeking help, and engaging with a hidden problem

#### Stereotyping and self-awareness of drinking

Most pharmacy staff reflected on regular experience of people with overt alcohol misuse in their day-to-day work and—along with other professional and PP participants—recognized a ‘park-bencher alcoholic’ stereotype of a person with alcohol misuse. However most participants believed many people with alcohol misuse do not fit this stereotype. Professional and PP participants described people who are unaware of how much they drink and/or what amount constitutes misuse as well as ‘self-aware drinkers’ i.e. people who recognize they drink ‘too much’ alcohol. Despite this insight, ‘self-aware drinkers’ were perceived to commonly be in denial about their drinking being a problem. Participants with lived experience of ArLD and some professionals saw this driven in part by social comparisons.

*‘you associated an alcoholic with being somebody on a park bench, drinking a bottle every single day. […] You think, I’m not because I’m not doing that.’ (C015/Patient/59F).*

#### Seeking advice and revealing hidden conditions

Many PP and clinician participants believed the asymptomatic development of ArLD perpetuated denial of alcohol as a problem. All participants regarded it universally known that drinking ‘too much’ can cause liver disease. Consequently ‘self-aware drinkers’ were believed to have some underlying concern about their liver. However, all participants with lived experience of ArLD recalled how the absence of symptoms—and a lack of awareness that ArLD may have developed despite this—allayed concerns.

Most participants perceived that people with alcohol misuse do not tend to seek advice for their drinking but some professional and PP participants believed the underlying concern about liver disease could motivate some ‘self-aware drinkers’ to take up an opportunity for a liver assessment, as described by a clinician who had worked at a liver charity event offering Fibroscans to the public.

‘They'll say, “I'd like to have a scan”, and when you get in the room […] “I'm aware that I drink quite a lot more than I should, […] but I haven't really done anything about it.”’ (C008/Clinician/52F)

#### Stigma, honesty and routinely contextualizing

Professional and PP participants recognized a need for healthcare professionals (HCPs) to routinely ask about alcohol with an ‘ask everyone’ approach perceived by some to avoid stigmatization through implied pre-judgement of an alcohol problem. This view was complicated by the perception of some professional and PP participants that many people with alcohol misuse do not want to speak about their drinking or will lie about it if asked. Postulated reasons for this included the aforementioned denial, potential personal consequences of revealing alcohol misuse (including stigmatization), and the communication style of the HCP asking. Most participants held the view that honest reporting of alcohol use was most achievable if asking routinely within a perceived relevant health context.

‘If I was in the chemist tomorrow, and they said, “Oh, we're doing this thing about alcohol, to see if you've got a liver problem. Would you be interested in answering a few questions,” or whatever, then I'd say, “Yes, that's fine.” […] but if the chemist just asked me out of the blue, “Hello, Mr X. How are you? Oh, how many pints…?” I'd think, well…’ (C020/Public/71M)

Concerns of offending and the potential taboo of alcohol conversations were recognised by many professional participants and seen as barriers to asking and advising about alcohol use for clinicians and pharmacy staff alike, with some clinicians perceiving this contributing to late diagnoses.

#### Enabling and facilitating motivated engagement

Regardless of how a person may be asked, the widely held view was that people need to be motivated to engage with any alcohol-related health advice or assessment. A goal for many professional participants was to generate motivation for patients to engage with care. In a pharmacy setting, offering and advertising an ArLD role as well as providing educational information about the risk of ArLD was believed essential for this. PP and professional participants also thought conveying the asymptomatic nature of ArLD to be important to enhance engagement.

Some participants conceptualized a pharmacy based ‘liver health check’ service, expecting this would incorporate a physical test. It was perceived by some PP and clinician participants that engagement of ‘self-aware drinkers’ would be motivated by, and possibly contingent on, getting such a test.

‘If it's just questions, there's probably little value in doing it […] to have something on a piece of paper that goes, actually we've done this blood test, and it's come back, and you need to be a bit careful, or you need to go now and see a GP, or a specialist, then that's valuable.’ (C019/Patient/44M)

It was also believed a ‘positive’ test for ArLD can motivate patients to engage with further care and reduce their alcohol consumption. However, a negative test was perceived by some clinicians and participants with lived experience of ArLD to potentially perpetuate current drinking habits, emphasizing the importance of educating about future risk.

### Professional roles, boundaries, and attributes

#### General health, alcohol and liver disease advice in community pharmacy

Both PP and professional participants regarded community pharmacists as qualified HCPs able to assess and advise on minor illnesses.

‘[pharmacists] they're trained professionals. They've got qualifications and knowledge. So it's like you would listen to your GP and you would listen to an NHS nurse. I would listen to them’ (C026/Public/56M)

Many pharmacy staff participants were motivated to provide health advice through being able to help pharmacy users and enjoying a role different to routine prescription-based work. Pharmacy staff experience of assessing and advising on alcohol use was mostly within advanced pharmacy services (e.g. hypertension case-finding service) or when delivering an alcohol identification and brief advice service, the latter only reported by three experienced staff (two pharmacists and one pharmacy assistant), each having worked for over 20 years’ in their roles.

Assessing and advising on alcohol use was seen by most participants to be within the capabilities of pharmacists. Views about pharmacy assistants were different; some PP participants perceived them insufficiently qualified whereas pharmacy staff participants believed that with appropriate communication skills training pharmacy assistants could engage pharmacy users in conversations about alcohol use. Despite these differing views, professional and PP participants acknowledged pharmacy assistants are typically the first (and often only) point of contact for pharmacy users and so would have to play a role.

‘Nine times out of ten, the pharmacist is actually behind a little counter. He can't see you. He's busy doing his drugs bit and it's the girls that come and see you. They're not medically qualified. Most of them aren't, anyway.’ (C015/Patient/59F)

Pharmacists (as well as many professional and PP participants) perceived that their existing work had already equipped them with the communication skills needed for potentially difficult conversations around alcohol use and ArLD. However, with the exception of one experienced pharmacist, pharmacy staff participants did not feel they currently had sufficient knowledge to appropriately assess and advise on alcohol use or ArLD but perceived this achievable with appropriate training. No participants were aware of ArLD being discussed in pharmacy. Moreover, pharmacy staff had little or no experience in discussing any liver disease.

#### Perceived abilities of community pharmacists for a role in ArLD identification

There was uncertainty whether pharmacists were sufficiently qualified to assess for and discuss ArLD. For some participants with lived experience of ArLD the presumption that ArLD has significant symptoms such as jaundice meant a doctor is needed and there is no role for a pharmacist. Additionally, ideas of assessment were often tied to expectations of a physical examination, blood test or scan and that these—as also acknowledged by pharmacy staff—are not routinely done by community pharmacists. Clinician participants also recognized this and whilst some perceived pharmacists able to be trained to conduct a liver test (Fibroscan or blood test), a lack of adequate space in most pharmacies, the time to do such a test and the cost of testing were believed to make this unfeasible. As such most professional and PP participants saw the role of a pharmacist to be that of finding and engaging people appropriate for testing and then referring for it, rather than conducting testing themselves.

‘I think, probably, [the pharmacists’] role would be more as that initial engagement, signposting on, educating, breaking down that first initial barrier of we have got people that can help you with this.’ (C005/Clinician/52F)

#### Bypassing GPs

Where a referral should be directed was considered by PP and clinician participants. The majority view was to bypass general practitioners (GPs) for a dedicated liver HCP, acknowledging healthcare structures may hinder this. This view was influenced by beliefs about GPs’ roles and abilities, in particular that they are not the best person for specialized advice, that GPs do not want to be gatekeepers to other services, and above all that it is increasingly difficult to get a GP appointment.

‘anything that's specific is usually outside their experience or their knowledge. Otherwise they'd have specialised in something. So they are a general practitioner, the first port-of-call, if you've got past triage of course’ (C020/Public/71M)

Clinician participants expressed concern about overwhelming the already stretched capacity in primary and secondary care if a pharmacy role was not planned in consideration of this. Clinician participants considering bypassing GPs did not believe this could be direct to a hospital consultant clinic and proposed alternatives of a community liver assessment service or a nurse-led clinic.

Views of bypassing GPs were accompanied with the expectation that a person’s GP still needs to be informed of any outcome—something paramount for two public participants who reported numerous chronic health conditions and saw their GP as central to their care. In contrast, both a public and clinician participant believed that for some pharmacy users the appeal of an assessment in pharmacy may be that their alcohol use is not shared with their GP.

#### Benefits and challenges of the community pharmacy setting

Pharmacies were described as widely accessible both in reference to geographic proximity and access to ‘walk-in’ healthcare advice without an appointment. Some professional participants also perceived community pharmacies a less stigmatizing location for people with alcohol misuse compared to a hospital or GP practice. This view was in part a result of experience of working with pharmacies to treat patients with hepatitis C.

In the context of repeat prescriptions, most participants perceived pharmacy users have more contact with pharmacy staff than their GP, meaning more opportunities for engagement. Additionally, this regular, familiar contact was seen an attribute for an ArLD role through reducing likelihood of causing offence and facilitating provision of ongoing support.

Privacy was regarded by all participants to be paramount and perceived attainable in pharmacies. However, staff time and capacity were considered a potential barrier. Many professional and PP participants also perceived most pharmacy users want to minimize time spent in pharmacy. As such, time efficiency of any role in ArLD was seen particularly important, perceived achievable through integration with other pharmacy services or by offering pharmacy users to attend at a pre-designated time.

‘everyday nowadays is short-staffed. […] even if you don't have prescription, you've got phone calls, you've got a customer coming in, so you hardly find time for it. That's why I was suggesting like if they had pre-booked it’ (C010/Assistant/32M)

#### Optimizing a service model of delivery in pharmacy

Professional participants envisaged a pharmacy ArLD role would be delivered as a service. Three aspects in particular were perceived by most pharmacy staff and some clinician participants to facilitate service delivery. These were: appropriate training for staff; strong external support; and remuneration at a level appropriate for the time required to deliver the service.

‘a service that will pay us £30 or £20 or whatever it is for the 15 minutes as opposed to me spending 15 minutes checking prescriptions I'll only be paid £3 for, my time is obviously better well spent providing the service. Service provision and remuneration is very important’ (C013/Pharmacist/50F)

Payment was also considered in relation to pharmacy users with the commonly expressed view that requiring pharmacy users to pay for a service themselves would deter engagement and one pharmacists believed it would exacerbate health inequalities.

### Communication, relationships, collaboration and support

#### Making referrals and pathways simple, clear and efficient

PP and professional participants described the potential use of secure email for referrals as part of an ArLD service and the importance of a dedicated referral form. For pharmacy staff this would provide a beneficial electronic audit trail and the ability to complete it at a time convenient to work demand.

Clinician participants also placed importance on pathways in ArLD being simple in terms of minimizing the number of patient-HCP visits required and maximizing what is delivered at each visit. This view was influenced by experiences that a proportion of patients disengage between each visit.

‘a patient with possible liver disease […] They can have several different interactions, in which the Swiss Cheese Model might make them get lost to follow up, so it's about trying to simplify it and streamline it for the patient, and also for the clinician’ (C004/Clinician/31F)

#### Two-way interprofessional communication

All professionals were unaware of any existing formalized routes of referral between pharmacies and clinical specialists. Pharmacy staff saw this reflective of the wider situation in community pharmacy, perceiving an absence of formalized communication routes to non-GP healthcare services and a reliance on signposting.

Regardless of communication route for any referral, pharmacy staff described only gaining knowledge of the outcome from pharmacy users themselves. Pharmacy staff enjoyed learning of benefits to pharmacy users of their referrals but some expressed frustration at the lack of direct feedback. They indicated that feedback could improve referral practices and, when concerned about a pharmacy user, alleviate worries about whether a pharmacy user received help.

‘Because you never get any feedback, you just hope for the best.’ (C002/Pharmacist/53M)

#### Establishing relationships and collaborating

For any collaborative working, many professional participants saw how establishing relationships between pharmacy staff and other HCPs was essential. These participants recognized difficulties in developing such relationships, with time to do so an evident barrier. Some also recognized how healthcare funding structures could inhibit collaborative relationships, as experienced in relation to flu vaccination services.

‘a pharmacy and the GP practice really fell out over [providing flu vaccinations]. There was a notice from the GP practice saying, “You must come to us,” and the pharmacy saying, “Actually, no, you don't”’ (C022/Public/54M)

Pharmacy staff and clinician participants who had established pharmacy-HCP relationships recognized their benefits in creating two-way communication channels, agreeing common goals, and enabling more effective care for patients. These were described in the context of engaging patients with Hepatitis C treatment, the hypertension case-finding service, and providing opioid substitution therapy.

#### Unmet support needs

Existing interprofessional collaboration in relation to wider support for people with alcohol misuse (with or without ArLD) was perceived an unmet need by professional participants. Existing ArLD pathways were viewed as ‘diagnosis-focused’ and not providing the holistic support patients may require to reduce their drinking, consequently reducing pathways’ effectiveness in preventing development or progression of ArLD.

‘you're giving advice for a patient to make the changes, but there isn't enough support around […] to make those changes[…] you do feel quite often with some of these pathways that you are just delaying the inevitable, rather than setting your patient up to succeed’ (C005/Clinician/52F)

In reflection of this unmet support need, many PP and professional participants perceived providing access to support to be important for any pharmacy role in ArLD care. The presence of existing relationships between some pharmacies and drug and alcohol services was perceived to facilitate this.

## Discussion

We examined a role for community pharmacists in identifying ArLD and therefore went beyond existing work describing screening for alcohol misuse in community pharmacies (Dhital et al. 2015; Hattingh and Tait 2018; Smith et al. 2023) Pharmacists are seen as qualified HCPs but testing for and diagnosing ArLD was mostly believed to be beyond their scope of practice. Instead, the role of community pharmacists in a care pathway for ArLD should be limited to identifying people at-risk, providing advice and referring on. In this role there is acknowledgement that the accessibility of pharmacies is an asset and an expectation the role should form a remunerated service. There was belief that referral onwards should avoid reliance on general practice but foster a strong collaborative with liver disease and alcohol support services.

Our participants’ perception that it is well known drinking ‘too much’ alcohol can cause liver disease corresponds to a 2017 survey of 2024 British adults in which 91% selected alcohol as something that causes or increases risk of liver disease (British Liver Trust 2017). ArLD being perceived in our study as a specific concern for some ‘self-aware drinkers’ contrasts with other qualitative research describing potential alcohol-related physical health impacts being considered a relative non-issue by people with alcohol misuse (Wallhed Finn et al. 2014). We recognize our different finding may reflect the focus of our study and participant selection, meaning greater consideration was given to ArLD as a health concern. Our finding that symptoms drive liver health concern and help-seeking supports other research showing that most people with alcohol misuse seek help when health problems become symptomatic (Simpson and Tucker 2002; Naughton et al. 2012). An absence of symptoms therefore has the potential to delay diagnosis and rationalizes initiatives to identify early asymptomatic disease—including community pharmacy assessment. Case-finding approaches that focus on people at risk of ArLD will concomitantly identify those who would benefit, and should be offered, alcohol interventions and support services known to be effective in reducing alcohol use (regardless of the presence of ArLD) (Kaner et al. 2018; Thursz et al. 2018). Subsequent reduction in alcohol use would reduce an individual’s risk of ArLD as well as more than 200 other alcohol-related health harms (Rehm and Shield 2019).

Participants in our study indicated that a ‘test’ for liver disease could increase engagement with a pharmacy ArLD service. The offer of a test for a specific condition in community pharmacy is known to boost engagement, e.g. pharmacy users undergoing a diabetic risk questionnaire and fingerprick blood glucose test had significantly higher uptake of subsequent referral to a GP than those undergoing the questionnaire alone (Krass et al. 2007). It may be that a physical test increases confidence in the result of an assessment, subsequently modifying engagement behavior (Krass et al. 2007; Saramunee et al. 2015).

The limited experience of alcohol advice being sought or provided in pharmacies in our study is expected. A 2017 study (Mackridge et al. 2017) found only 5% of pharmacies in England provided an alcohol screening and intervention service. However, our study supports other research indicating the public generally perceive community pharmacists appropriate and capable to assess and advise on alcohol use (Sheridan et al. 2012; Fitzgerald et al. 2015; Mackridge et al. 2016). Our study adds new insights in that clinicians involved in the care of patients with ArLD share this view.

Our findings indicate uncertainty about capabilities of pharmacies in diagnosis and further management of ArLD. A systematic review of PP perceptions of pharmacists described similar concerns (Hindi et al. 2018). As such, the described desire to bypass GPs expressed by participants in our study emphasizes the importance of interprofessional collaboration between pharmacists and clinicians in liver services to enable a pharmacist role in identifying ArLD. Such collaboration has been seen as a key factor in the success of community pharmacy hepatitis C screening and treatment (Radley et al. 2020; Klepser et al. 2022).

### Limitations

Our study has limitations. We recruited pharmacy staff from independent, small and large chain pharmacies but not from supermarket-based pharmacies nor any of the three largest pharmacy chains in England (NHS Business Services Authority 2024). However, we do not believe this a major limitation given the majority of pharmacies in England are small chain or independent and that these are preferred by the public (Duxbury and Fisher 2022; NHS Business Services Authority 2024). Need and expectation of appropriate remuneration for a pharmacist role in ArLD described in our study reflects wider research recognizing remuneration as a key feature for the success of a pharmacy service (Weir et al. 2019) However, many countries’ health systems do not provide remuneration for such services (Hussain and Babar 2023) and as such our study’s findings may not apply to such settings.

Participants were either working in healthcare or recruited via healthcare services and so findings may not reflect those who do not currently access healthcare. However, many participants had either personal experience of not accessing healthcare or working with people who don’t tend to, which we believe will have increased relevance of findings to this population. Recruited pharmacy staff may have been those more interested in, and positive about, expanding pharmacy roles. The potential for such selection bias is well recognized in qualitative research but we believe we reduced this through recruiting multiple different stakeholders as well as specifically exploring perceived challenges to a pharmacist role in ArLD in the interviews (Kaae and Traulsen 2020). Lastly, participants were considering a hypothetical role for pharmacists. We encouraged participants to reflect on their own experiences to provide experience-informed views but recognize further feasibility work in which a role is delivered, rather than conceptualized, is needed to gain further understand and fully develop a pharmacist role in identifying ArLD.

## Conclusion

Relevant stakeholders recognized a potential role for community pharmacists in identifying ArLD, with the focus being on finding people at risk and engaging them with care. To be realized, a collaborative approach with liver and support services is essential, with access to community based liver testing an anticipated requirement. Coupled with the increasing drive for pharmacists to be a first port of call for illness in the community, a pharmacist role in ArLD identification could increase awareness and enable earlier diagnosis and subsequent care for ArLD and alcohol misuse in people who may not access healthcare elsewhere.

## Data Availability

The data underlying this article cannot be shared publicly for the privacy of individuals that participated in the study. The data will be shared on reasonable request to the corresponding author.
